# CT Findings in a Novel Coronavirus Disease (COVID-19) Pneumonia at Initial Presentation

**DOI:** 10.1155/2020/5436025

**Published:** 2020-08-15

**Authors:** Chao Xiang, Ji Lu, Jun Zhou, Li Guan, Cheng Yang, Changzhu Chai

**Affiliations:** ^1^The First College of Clinical Medical Science, China Three Gorges University, Yichang, Hubei, China 443000; ^2^Department of Radiology, Yichang Central People's Hospital, Yichang, Hubei, China 443000; ^3^Department of Ultrasound, Yichang Central People's Hospital, Yichang, Hubei, China 443000; ^4^Department of Respiratory Medicine, Yichang Central People's Hospital, Yichang, Hubei, China 443000

## Abstract

**Background:**

COVID-19 first broke out in China and spread rapidly over the world.

**Objectives:**

To describe the CT features of COVID-19 pneumonia and to share our experience at initial diagnoses. *Patients and Methods*. Data from 53 patients (31 men, 22 women; mean age, 53 years; age range, 16-83 years) with confirmed COVID-19 pneumonia were collected. Their complete clinical data was reviewed, and their CT features were recorded and analyzed.

**Results:**

The average time between onset of illness and the initial CT scan was six days (range, 1-42 days). A total of 399 segments were involved and distributed bilaterally (left lung: 186 segments [46.6%], right lung: 213 segments [53.4%]) and peripherally (38 [71.7%] patients). Multiple lobes (45 [84.9%]) and bilateral lower lobes (left lower lobe: 104 [26.1%], right lower lobe: 107 [26.8%], and total: 211 [52.9%]) were the most commonly involved. Ground-glass opacity with consolidation (24 [45.3%]) and pure ground-glass opacity (28 [52.8%]) were the main findings. The other findings were crazy-paving (14 [26.4%]), bronchiectasis (12 [22.6%]), atelectasis (7 [13.2%]), parenchymal bands (6 [11.3%]), air bronchogram (6 [11.3%]), interlobular thickening (5 [9.4%]), reticular pattern (1 [1.9%]), and pleural effusion (1 [1.9%]).

**Conclusions:**

Most COVID-19 pneumonia patients had abnormalities on chest CT images at initial presentation. Imaging features combined with patient's exposure history and onset symptoms could facilitate the identification of the suspected patient for further examinations.

## 1. Introduction

An epidemic novel coronavirus infection broke out in Wuhan (the capital city of Hubei Province in central China) in December 2019. The infected people were initially considered highly associated with exposure to the Huanan seafood wholesale market. From December 31, 2019, to January 3, 2020, a total of 44 patients with pneumonia of unknown etiology were reported to WHO [1]. As this infection broke out during the spring-festival travel rush, it spread rapidly to all provinces of China, and cases were subsequently found in 26 countries worldwide [2]. As of March 25^th^ 2020, 372,757 confirmed cases and 16,231 deaths were reported globally [3].

On January 7, 2020, a new type of coronavirus (SARS-CoV-2) was isolated by the Wuhan Institute of Virology at the China Academy of Sciences. SARS-CoV-2 was identified as the causative virus by Chinese authorities on January 7, 2020. This novel coronavirus had not been previously identified in humans and was tentatively named the 2019 novel coronavirus (2019-nCoV) by WHO on January 13, 2020, and was officially named COVID-19 on February 11, 2020 [4].

COVID-19 leads to respiratory infections similar to those of SARS and MERS, causing pneumonia, severe acute respiratory syndrome, kidney failure, and even death. Data have suggested that the SARS-CoV-2 is generally less pathogenic than SARS-CoV and is much less pathogenic than MERS-CoV [5]. Due to previous reported cases which are biased to more severe cases at the early stages of the epidemic, the death rate was relatively high [5]. But the true mortality risk might be much lower, and it was reported that the death rate caused by pneumonia-related disease is about 3% [5]. The pathological findings of COVID-19 have indicated that it is greatly similar to those seen in SARS and MERS coronavirus infections [2].

CT is an important tool for identifying the infected patients, and it is helpful for the follow-up evaluation of the treatment. Although the CT features of COVID-19 pneumonia have been reported in the literature, none of them have excluded patients with basic lung disease (such as COPD, pulmonary tuberculosis, and interstitial lung disease) in their studies. These diseases may have the same features and patterns that are similar to those caused by COVID-19 pneumonia. Here, we aim to analyze and discuss the CT features of COVID-19 pneumonia in patients without severe basic pulmonary disease and share our experience in CT diagnoses.

## 2. Materials and Methods

The patients' clinical and image data used in this study were approved by the review board of our institution, and the patients' privacy was well protected. Informed consent was waived by the review board for this retrospective study.

### 2.1. Patients

**Figure 1:**
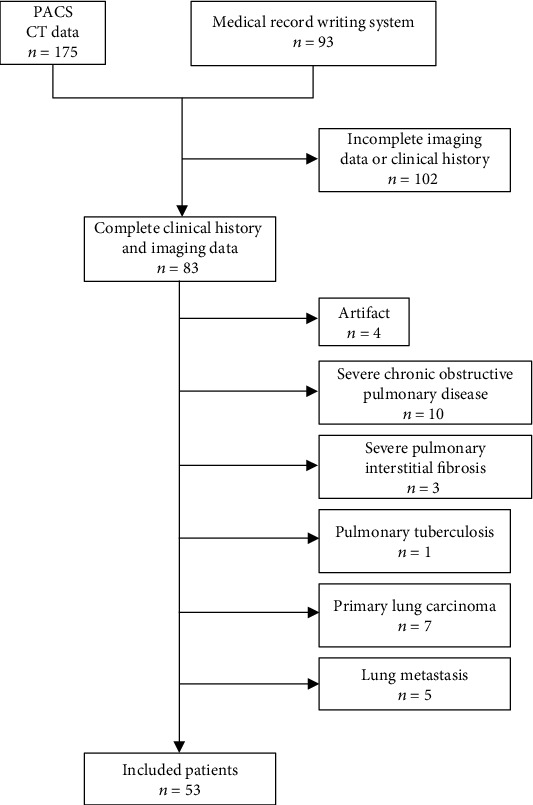
Flow diagram of patient selection.

Between December 2019 and February 20, 2020, 185 COVID-19-infected patients were confirmed at the First College of Clinical Medical Science, China Three Gorges University, and the Yichang Central People's Hospital. Some patients (*n* = 102) were excluded for the following reasons: (a) respiratory motion artifacts on CT images, (b) incomplete clinical and/or imaging data, (c) severe basic pulmonary disease (including severe pulmonary interstitial fibrosis, severe chronic obstructive pulmonary disease, pneumoconiosis, and pulmonary tuberculosis), and (d) primary or secondary pulmonary tumor. After the selection, 53 eligible patients (31 males, mean age: 53 ± 16 years, range: 16-83 years) were included in our study (Figure 1).

### 2.2. Review of Chest CT Scans and Images

All patients underwent nonenhanced chest CT scanning (UCT, United Imaging, Shanghai, China; Ingenuity Flex, Philips, Netherland) at initial presentation. The scanning parameters were as follows: supine position, thickness: 2 mm or 5 mm, interval: 5 mm, tube voltage: 130 kV, tube current: 233 mA, scanning during inspiration, and 1 second per scan.

All imaging data were transferred to picture archiving and communication systems (PACS, United Imaging, Shanghai, China) and reviewed independently by two radiologists (C.X. and J.L.) with more than 10 years of experience in thoracic CT image interpretation. The location, distribution, morphology, CT features, and patterns of the lesions were recorded and analyzed. Disagreements were resolved by consensus.

The CT image characteristics were recorded as follows: (a) lesion's location (segment), (b) morphology (patchy, nodular, and linear), (c) distribution (single or multiple, peripheral or/and central), (d) type (ground-glass opacity, consolidation, and linear opacity), (e) pattern (reticulation, parenchymal bands, crazy-paving, and interlobular thickening), (f) atelectasis, (g) cavitation, (h) pleural effusion, (i) hilar or mediastinal lymphadenopathy, (j) bronchiectasis, and (k) air bronchogram. Ground-glass opacity was defined as hazy increased opacity in the lung that did not obscure the bronchial and vascular architecture. Consolidation was defined as homogeneously increased opacity in the lung without clear margins of vessels and bronchus. Peripheral distribution was defined as the lesion located at the outer one-third of the lung; otherwise, it was defined as central. Other abnormalities, if any, were noted.

### 2.3. Diagnostic Criteria

We used the diagnosis and treatment protocols for COVID-19 pneumonia (6^th^ edition) promulgated by the National Health Commission of the People's Republic of China [6]. The suspected cases comprised any one of the criteria in epidemiology and any two of the criteria in clinical manifestations or any three of clinical manifestations with uncertain epidemiological history. The epidemiology and clinical manifestations were as follows:

#### 2.3.1. Epidemiology

1. History of travel to or resident in Wuhan or exposure to patients with respiratory symptoms or/and fever from Wuhan within 14 days before the onset of illness
2. Close contact with COVID-19-confirmed pneumonia patients
3. A cluster outbreak (community or nosocomial) of COVID-19 pneumonia

#### 2.3.2. Clinical Manifestations

1. Fever and/or respiratory symptoms
2. CT features of viral pneumonia
3. Normal level of total leukocytes or leukopenia and lymphocytopenia at an early stage. Confirmed cases were diagnosed with real-time fluorescence polymerase chain reaction (RT-PCR) or high origin of similarity with SARS-CoV-2 by next-generation sequencing

## 3. Results

Fifty-three COVID-19-infected patients(31 males, mean age: 53 ± 16 years, range: 16-83 years; average temperature: 38.0 ± 0.5°C [100.4 ± 32.9°F]) were included in our study. Seven patients (13.2%) (including three asymptomatic patients) had exposure history, while 46 patients (86.8%) (including two asymptomatic patients) had no clear exposure history. Onset symptoms and morbidity were listed in Table 1. Fever (66.0%), cough (39.6%), fatigue (18.9%), myalgia (15.1%), and expectoration (15.1%) were the main onset symptoms. The average time between onset and the initial CT scan was six days, range from 1 to 42 days. All the patients were confirmed to have COVID-19 infection via RT-PCR.

**Figure 2:**
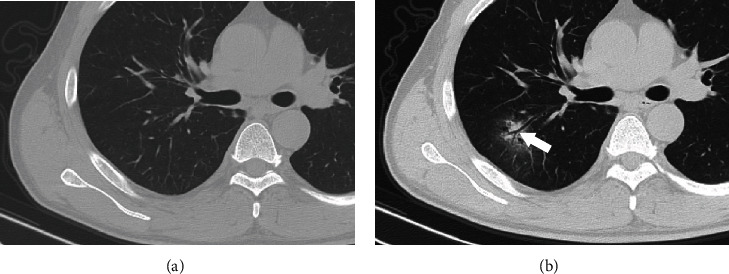
A 51-year-old man with unknown exposure history presented with fever and dry cough for 2 days. (a) The first chest CT scan shows normal findings. (b) Four days later, the second chest CT scan shows patchy ground-glass opacity with consolidation in the posterior segment of the right upper lobe. Bronchiectasis (arrow) could be seen in the lesion.

Of the 53 patients, three patients (5.7%) showed normal findings on the first thoracic CT scan, but their second CT scan manifested abnormal changes (Figure 2). After reviewing initial CT image data, a total of 399 involved segments were found, including 186 segments (46.6%) in left lobes and 213 segments (53.4%) in the right lobes. In the right upper lobe, 22 lesions (5.5%) were located in the anterior segment, 30 (7.5%) in the posterior segment, and 18 (4.5%) in the apical segment. In the right middle lobe, 14 lesions (3.5%) were seen in the medial segment and 22 (5.5%) in the lateral segment. In the right lower lobe, 8 lesions (2%) were seen in the medial basal segment, 18 (4.5%) in the anterior basal segment, 30 (7.5%) in the lateral basal segment, 16 (4%) in the superior segment, and 35 (8.8%) in the posterior basal segment. In the left upper lobe, there were 22 lesions (5.5%) in the apicoposterior segment, 24 (6.0%) in the anterior segment, and 36 (9%) in lingual segment (including the superior and inferior segments). In the left lower lobe, there were 11 lesions (2.8%) in the medial basal segment, 24 (6%) in the anterior basal segment, 24 (6%) in the lateral basal segment, 17 (4.3%) in the superior segment, and 28 (7%) in the posterior basal segment (Table 2).

Multiple lobes were involved in 45 patients (84.9%), and bilateral distribution was found in 45 patients (84.9%). Eight patients (15.1%) involved a single lobe, and of these patients, 3 (5.7%) had a single lesion involving a single segment (two located at the posterior segment of the right upper lobe and one at the anterior basal segment of the left lower lobe). Bilateral lower lobes were the most commonly involved (left lower lobe: 104 [26.1%], right lower lobe: 107 [26.8%], and total lower lobe: 211 [52.9%]). Of the involved lower lobes, the anterior basal segment (left: 24 [6%], right: 18 [4.5%]), lateral basal segment (left: 24 [6%], right: 30 [7.5%]), and posterior basal segment (left: 28 [7%], right: 35 [8.8%]) were the most commonly affected segments. Lesions distributed in the peripheral zone were seen in 38 patients (71.7%), and 15 lesions (28.3%) were found in both the peripheral and central affected zones.

**Figure 3:**
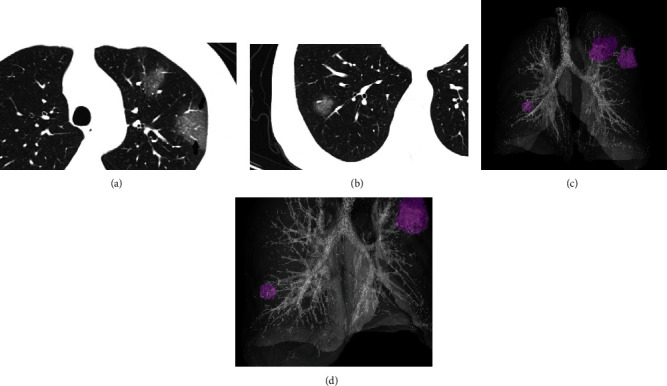
A 54-year-old man presented with fever for one day. The initial CT scan shows multiple patchy ground-glass opacities located at the anterior segment and apicoposterior segment of the left upper lobe (a) and lateral segment of the right lower lobe (b) with thickened intralesional vessels (black arrow). The 3D reconstruction (c, d) shows bilateral, multilobar, and peripheral distribution (purple nodules).

**Figure 4:**
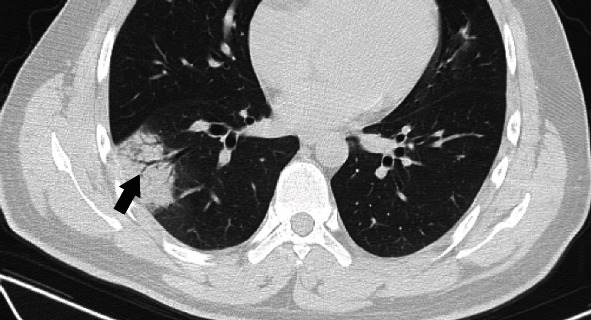
Imaging in a 26-year-old man who presented with fever for 6 days. Axial CT image shows patchy consolidation with air bronchogram (black arrow) in the lateral basal segment of the right lower lobe.

Twenty-four (45.3%) patients showed ground-glass opacity with consolidation (Figure 2), 28 patients (52.8%) showed pure ground-glass opacity (Figure 3), and only 1 patient (1.9%) showed pure consolidation (Figure 4).

**Figure 5:**
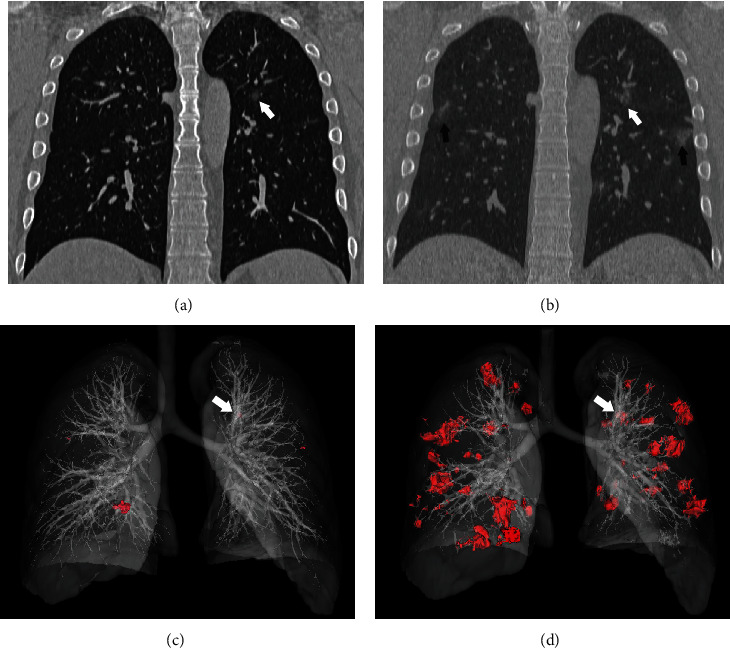
Imaging in a 25-year-old man who presented with fever and cough for 2 days. The coronal CT image shows a small ground-glass nodule (white arrow) in the apicoposterior segment of the left upper lobe (a). Three days later, the second CT image shows multiple nodular and patchy ground-glass opacities (black arrow) distributed bilaterally and peripherally. The volume of preexisting ground-glass opacities (white arrow) is increased (b). 3D reconstruction (c, d) shows the intrapulmonary changes during the time of the two CT scans.

**Figure 6:**
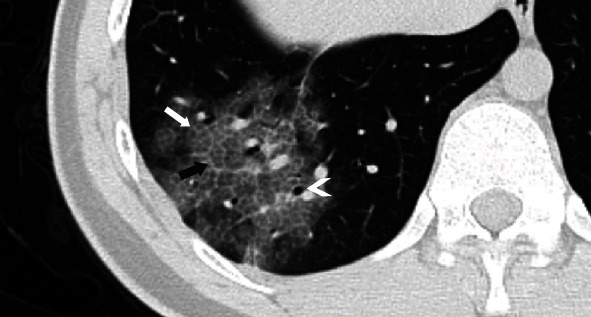
A 16-year-old boy presented with fever for 5 days. The axial CT image shows patchy ground-glass opacities with interlobular (black arrow) and intralobular (white arrow) septum thickening, showing a crazy-paving pattern. Bronchiectasis is also manifested (arrowhead).

**Figure 7:**
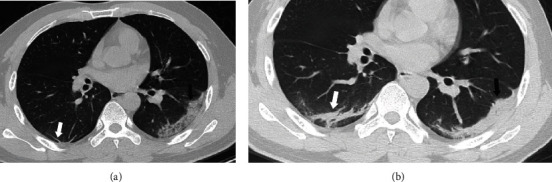
A 44-year-old man presented with fever, vomiting, nausea, and fatigue for 7 days. Axial CT image (a) shows patchy ground-glass opacities with focal consolidation (black arrow) in the left lower lobe. Parenchymal band is also manifested (white arrow) in the right lower lobe. Three days later, the second CT scan (b) shows increased attenuation of the lesion in the left lower lobe (black arrow) and thickening of the lesion in the right lower lobe (white arrow).

**Figure 8:**
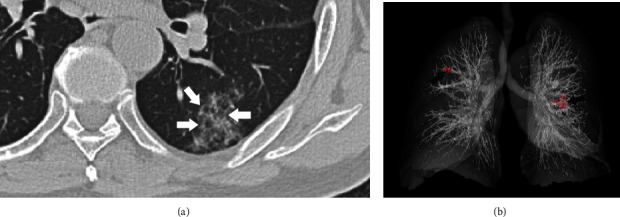
A 76-year-old man presented without onset symptoms. Axial CT image shows patchy ground-glass opacities located at the lateral basal segment of the left lower lobe (a). The interlobular septa (white arrow) are thickened and show a reticular pattern. The 3D reconstruction image shows bilateral opacities (red spot and black arrow) with peripheral distribution (b).

Of the 53 patients, 48 (90.6%) demonstrated lesions with patchy morphology (Figure 4), and 11 patients (20.8%) were nodular (Figure 5). A crazy-paving pattern (Figure 6) was demonstrated in 14 patients (26.4%), parenchymal bands (Figure 7) in 6 patients (11.3%), air bronchogram (Figure 4) in 6 patients (11.3%), interlobular thickening (Figure 6) in 5 patients (9.4%), reticular pattern (Figure 8) in 1 (1.9%) patient, and pleural effusion in 1 (1.9%) patient. Bronchiectasis was found in 12 (22.6%) patients. Of the 12 patients, three demonstrated traction bronchiectasis. There was no evidence for hilar and mediastinal lymphadenopathy or cavitation (Table 3).

## 4. Discussion

COVID-19 is a new viral infection in humans caused by severe acute respiratory syndrome coronavirus 2 (SARS-CoV-2) in the Coronaviridae family. Two strains of coronavirus, SARS and MERS, emerged in China and the Middle East in 2002 and 2017, respectively, causing a worldwide alert by WHO. This time, COVID-19 broke out in China and alerted the world once more. Most of COVID-19 pneumonia patients have abnormal changes on chest CT images at initial CT scan, but whether these changes had existed prior to being infected is unknown, because patients with severe basic pulmonary disease have not been excluded in previous studies. In the present study, we excluded patient with severe basic pulmonary diseases to minimize the overlap features. CT is an important tool for identifying SARS-CoV-2-infected patients. Ground-glass opacity with or without consolidation combined with bilateral, multilobe, and peripheral involvement of the lung was the main feature and distribution of COVID-19 pneumonia.

Previous researches showed that which host is susceptible to SARS-CoV infection is mostly decided by the affinity between host angiotensin-converting enzyme 2 (ACE2) and the viral receptor-binding domain (RBD) [7]. The recent studies show that the genome sequence of SARS-CoV-2 is quite similar to that of SARS-CoV but distant from MERS-Cov [8–10]. These findings indicate the following: (1) ACE2 is used as a receptor by SARS-CoV-2, which is similar to SARS-CoV; (2) SARS-CoV-2 can infect the human cell; and (3) SARS-CoV-2 can spread from person to person [7].

ACE2 is expressed in human airway epithelia, lung parenchyma, and the gastrointestinal tract, which are the major sites of replication of the virus [11, 12]. The lower respiratory tract symptoms (including fever, cough, and shortness of breath) are the major onset symptoms and are similar to those caused by SARS-CoV [7, 13]. Diarrhea was also reported in the literature [14], and there was one patient (1.9%) in our group. Pan et al. reported that infected individuals can be infectious prior to the onset of symptoms [15]. In our group, 5 patients (9.4%) were asymptomatic at initial presentation but confirmed by viral nucleic acid test. This suggested that clinical symptoms are not essential components for identification or diagnosis.

There was a slight predilection for males (male: 31 [58.5%]) and old age (>60 years in 30 [56.6%] patients) in our cohort, and this is similar to previous findings [16]. However, other studies showed no obvious sex predilection [17, 18]. This could be explained by different demographic features and the small size of our group.

Chest radiographs are less sensitive than a CT scan in detecting small lesions; thus, the CT scan is the first choice for initial identification [19]. Most types of COVID-19 pneumonia have abnormal radiographic changes at initial presentation, and CT features and patterns are similar to those of viral pneumonia. Symptomatic patients without radiographic changes have been reported in the literature [18, 20], and these patients are also found in our group (3 [5.7%]), suggesting that there is an incubation period (1-14 days) prior to positive findings of the CT scan. Negative findings of the CT scan could not exclude the infected patients.

Multilobe, multisegment, and peripheral-zone involvement was very common in our study. Chung et al. reported that, of 21 COVID-19 pneumonia patients, 20 had multiple affected lobes, and of 18 patients with lung opacity, 16 (88.9%) had bilateral distribution [20]. Shi et al. reported that 79% of patients had bilateral lung involvement and 54% of patients showed peripheral distribution [17]. In our cohort, the involvement of 399 segments (left lobe: 186 [46.6%] seg, right lobe: 213 [53.4%] seg) at initial CT scan distributed bilaterally. The lower lobes (especially the anterior basal segment, lateral basal segment, and posterior basal segment) are the most commonly affected sites. This may be because of the anatomical structure of the trachea and bronchi—the bronchus of lower lobes is relatively straight, and the virus arrives more easily in the lower lobes [17]. Here, 46 (86.8%) of our cases show multiple lobe involvement with lower lobe predominance. Only 8 patients (13.2%) had infected single lobes, and of the 8 patients, 3 patients involved a single segment. Our study shows that the distribution of lesions preferred to affect the peripheral zone, which is similar to those in a radiological study with SARS and MERS [21, 22]. This might be due to the viral capability of reaching the terminal bronchioles and alveoli. The extensive bilateral lung involvement, like SARS-CoV, might be consistent with high initial viral loads [15, 23].

Ground-glass opacity with or without consolidation are main features of the disease. These features are highly suggestive of acute interstitial pneumonia of the disease and are consistent with its histopathological findings that COVID-19 pneumonia involves both parenchyma and interstitial lung tissue [2]. These features could also be found in SARS and MERS. Pure consolidation opacity at an initial CT scan is rare.

Other findings include interlobular and intralobular septum thickening, crazy-paving pattern, reticular pattern, air bronchogram, atelectasis, and bronchiectasis. When an inflammatory response occurred, lung macrophages reside in the lung interstitium, and alveoli play critical roles in initiating and maintaining inflammation [23]. A histopathological report of COVID-19 pneumonia showed that lung tissue displayed pulmonary edema with hyaline membrane formation and lymphocytes predominated the infiltration of interstitial mononuclear inflammatory and alveolar damage with cellular fibromyxoid exudates [2]. These features are similar to those of SARS and MERS [24, 25]. These pathological findings are consistent with the features of ground-glass appearances, consolidation, interlobular and intralobular septum thickening, and crazy-paving pattern. The crazy-paving pattern was originally reported in patients with alveolar proteinosis. This pattern also could be found in other pulmonary diseases (such as usual interstitial pneumonia, pulmonary edema, and adult respiratory distress syndrome) that affect both the interstitial and airspace compartments [26, 27]. The reticular pattern is associated with intralobular lines or interlobular septum thickening indicating interstitial changes (interstitial inflammation or fibrosis).

Parenchymal bands were found in 6 (11.3%) patients. This reflects fibrosis and distortion of the lung architecture [26]. Fibrosis may demonstrate in the late stage of SARS or can be due to steroid therapy [28]. Parenchymal bands manifested at the early CT images suggested the preexisting obsolete lesions. If the alveolar collapsed, then segmental atelectasis could be seen on CT images with a thick parenchymal band pointing to the hilus.

An air bronchogram can be seen within ground-glass lesions and consolidation, suggesting unobstructed proximal airways. Bronchiectasis is associated with dilatation of bronchioles suggesting fibrotic changes within the lesion. Pleural effusion and lymphadenopathy were reported in the literature [17], but they are rare features of SARS and MERS; only 1 patient showed pleural effusion in our cohort. It was hard to say if these changes were caused by SARS-CoV-2 infection because comorbid patients were not excluded in other studies, and iatrogenic reasons cannot be excluded either. No evidence of cavitation was seen, and the result is similar to recent literature and SARS and MERS pneumonia [20, 27, 29].

It is noticeable that, of the 5 asymptomatic infected patients, 3 patients had a clear exposure history. Thus, an epidemiological survey of a patient is very important in the initial identification of a suspected patient. If a clear exposure history is obtained, then the patient should be quarantined for further examination. Unlike patients in Wuhan city, most our patients do not have a clear exposure history. Thus, during the outbreak, a patient with symptoms of low respiratory infection should be isolated for chest CT scan and nucleic acid testing. Repeat CT scan and nucleic acid testing might be done, because the negative result of both the CT scan and nucleic acid testing can be obtained at the initial time.

In our experience, exposure history is the most important clue for identifying high-risk individuals. Clinical diagnosis should combine with the patient's symptom, CT changes, and exposure history. None of these components could be used for diagnosing COVID-19 alone. Although a patient with exposure history may be asymptomatic and obtained negative results of CT findings and viral nucleic acid test at initial presentation, the potential infection cannot be totally excluded, and performing repeating CT scan and coronavirus RNA test is needed.

Our study had several limitations. First, we had a small cohort, performed a single-center study, and excluded the comorbid patients. This may lead to selection bias. Second, some of our patients had a CT scan with a thickness of 5 mm. This might overlook subtle changes in the lesion.

In conclusion, COVID-19 infection appears clinically milder than SARS or MERS in terms of severity and fatality, but stronger in terms of transmissibility. An exposure history is extremely important for identifying high-risk groups for quarantine and further examination, even if they are asymptomatic. Chest CT scans are helpful in identifying the suspected patient, even though negative results may be obtained during the incubation period. If the features are consistent with viral pneumonia, then a viral nucleic acid test should be done, and these patients should be admitted to a hospital for isolated observation and further examination. Ground-glass opacity and consolidation with multiple, bilateral, and lower lobe distribution are the main features of COVID-19 pneumonia at initial CT scan. Other CT findings are crazy-paving, bronchiectasis, air bronchogram, and atelectasis, which are similar to features found in SARS and MERS, and these features and patterns are nonspecific for diagnosing COVID-19 pneumonia. Viral nucleic acid test is the golden standard for confirmation, but it has high specificity for identifying COVID-19 infection and relatively low sensitivity. Thus, negative results will not exclude the high-risk group; repeated nucleic acid testing should be done.

## Figures and Tables

**Table 1:** Patients' demographic and characteristics.

Demographic and characteristics	Number
Gender	
Men	31 (58.5%)
Women	22 (41.5%)
Mean age (y)	53 ± 16 (range: 16-83)
Exposure history	7 (13.2%)
No exposure history	46 (86.8%)
Symptoms at initial presentation	
Fever	35 (66.0%)
Cough	21 (39.6%)
Myalgia	8 (15.1%)
Expectoration	8 (15.1%)
Sore throat	4 (7.5%)
Nausea and/or emesis	3 (5.7%)
Shortness of breath	4 (7.5%)
Fatigue	10 (18.9%)
Headache	5 (9.4%)
Chills	2 (3.8%)
Diarrhea	1 (1.9%)
No symptoms	5 (9.4%)
Average temperature	38.0 ± 0.5°C (100.4 ± 32.9°F)

**Table 2:** Segmental involvement at initial CT scan.

Location	No. of segments
Left lung	186 (46.6%)
Right lung	213 (53.4%)
Right upper lobe	70 (17.5%)
Anterior segment	22 (5.5%)
Posterior segment	30 (7.5%)
Apical segment	18 (4.5%)
Right middle lobe	36 (9%)
Medial segment	14 (3.5%)
Lateral segment	22 (5.5%)
Right lower lobe	107 (26.8%)
Superior segment	16 (4%)
Medial basal segment	8 (2%)
Anterior basal segment	18 (4.5%)
Lateral basal segment	30 (7.5%)
Posterior basal segment	35 (8.8%)
Left upper lobe	82 (20.6%)
Apicoposterior segment	22 (5.5%)
Anterior segment	24 (6.0%)
Lingular segment	36 (9%)
Left lower lobe	104 (26.1%)
Superior segment	17 (4.3%)
Medial basal segment	11 (2.8%)
Anterior basal segment	24 (6%)
Lateral basal segment	24 (6%)
Posterior basal segment	28 (7%)

**Table 3:** Characteristics of CT findings.

CT findings	No. of patients
Distribution	
Multilobe	45 (84.9%)
Bilateral	45 (84.9%)
Type of the lesion	
Pure ground-glass opacity	28 (52.8%)
Ground-glass opacity with consolidation	24 (45.3%)
Pure consolidation	1 (1.9%)
Morphology of the lesion	
Patchy	48 (90.6%)
Nodular	11 (20.8%)
Patterns	
Crazy-paving	14 (26.4%)
Parenchymal bands	6 (11.3%)
Air bronchogram	6 (11.3%)
Interlobular thickening	5 (9.4%)
Reticulation	1 (1.9%)
Bronchiectasis	12 (22.6%)
Atelectasis	7 (13.2%)
Other findings	
Pleural effusion	1 (1.9%)
Lymphadenopathy	0
Cavitation	0

## Data Availability

The data may be available upon email request.

## Abbreviations

COVID-19: Coronavirus disease 2019
SARS: Severe adult respiratory syndrome
SARS-CoV: Severe adult respiratory syndrome coronavirus
MERS: Mideast respiratory syndrome
MERS-CoV: Mideast respiratory syndrome coronavirus
PACS: Picture archiving and communication system
RT-PCR: Real-time fluorescence polymerase chain reaction
AEC2: Angiotensin-converting enzyme 2
RBD: Receptor-binding domain
RNA: Ribonucleic acid.
